# SIRT2 is required for efficient reprogramming of mouse embryonic fibroblasts toward pluripotency

**DOI:** 10.1038/s41419-018-0920-3

**Published:** 2018-08-30

**Authors:** Ah-Young Kim, Eun-Mi Lee, Eun-Joo Lee, Jae-Hong Kim, Kyoungho Suk, Eunhye Lee, Keun Hur, Yean Ju Hong, Jeong Tae Do, SunYoung Park, Kyu-Shik Jeong

**Affiliations:** 1 Department of Pathology, College of Veterinary Medicine, Kyungpook National University, Daegu, 41566 Republic of Korea; 2 Stem Cell Therapeutic Research Institute, Kyungpook National University, Daegu, 41566 Republic of Korea; 3 Department of Phamacology, Brain Science & Engineering Institute, BK21 Plus KNU Biomedical Convergence Program for Creative Talent, School of Medicine, Kyungpook National University, Daegu, 41944 Republic of Korea; 4 Department of Biochemistry and Cell Biology; and BK21 Plus KNU Biomedical Convergence Program for Creative Talent, Department of Biomedical Science, School of Medicine, Kyungpook National University, Daegu, 41944 Republic of Korea; 5 Department of Stem Cells and Regenerative Biology, College of Animal Bioscience and Technology, Konkuk University, Seoul, 05029 Republic of Korea

## Abstract

The role of sirtuins (SIRTs) in cancer biology has been the focus of recent research. The similarities between underlying pathways involved in the induction of pluripotent stem cells and transformation of cancer cells revealed the role of SIRTs in cellular reprogramming. Seven SIRTs have been identified in mammals and downregulation of SIRT2 was found to facilitate the generation of primed pluripotent stem cells, such as human induced pluripotent stem cells. Herein, we evaluated the role of SIRT2 in naive pluripotent stem cell generation using murine cells. We found that absolute depletion of SIRT2 in mouse embryonic fibroblasts resulted in a notable reduction in reprogramming efficiency. SIRT2 depletion not only upregulated elements of the INK4/ARF locus, which in turn had an antiproliferative effect, but also significantly altered the expression of proteins related to the PI3K/Akt and Hippo pathways, which are important signaling pathways for stemness. Thus, this study demonstrated that SIRT2 is required for cellular reprogramming to naive states of pluripotency in contrast to primed pluripotency states.

## Introduction

Sirtuins (SIRTs) are highly conserved NAD^+^-dependent deacetylases^1^. In mammals, there are seven different SIRTs (SIRT1–SIRT7) with discrete subcellular localizations and distinct functions^2^. SIRT1, SIRT6, and SIRT7 are mainly located in the nucleus, SIRT2 is mainly in the cytoplasm, and SIRT3, SIRT4, and SIRT5 are localized to the mitochondria^3^. Because SIRTs play a key role in maintaining genomic integrity by coordinating cellular responses to various stresses, their aberrant regulation causes tumorigenesis^4^.

According to previous studies, overlapping mechanisms control induced pluripotent stem cell (iPSC) production and tumorigenesis^5,6^. A study comparing the transcriptomes of iPSCs and oncogenic foci (a tumor cell mass created in vitro) from common parental fibroblasts revealed many similarities^7^. Thus, pluripotency and tumorigenicity appear to be closely associated; consequently, SIRTs may be related to cellular reprogramming.

Several reports have described a correlation between SIRTs and iPSC reprogramming efficiency. SIRT1 not only enhances iPSC generation through p53 deacetylation, but also is required for proficient post-reprogramming telomere elongation^8,9^. Because SIRT1 is the closest mammalian homolog of yeast Sir2, it has been the most extensively studied SIRT in mammals. Other SIRTs (SIRT2–SIRT7) have received less attention in this regard; a previous study revealed that SIRT6 improves iPSC reprogramming efficiency in aged human dermal fibroblasts by regulating miR-766 transcription^10^. Another study showed that pluripotency genes are upregulated by silencing of SIRT3 in bovine fibroblasts; however, the exact role of SIRT3 in iPSC reprogramming remains unclear^11^.

SIRT2 is primarily found in the cytoplasm where it transiently localizes to the nucleus during the G2/M phase. As a class III histone deacetylase, it deacetylates histone H4 at lysine 16 upon migration to the nucleus^12^. Thus, SIRT2 has been mainly studied for its role in regulating mitosis^13,14^. Because cancer is a consequence of uncontrolled cell division and proliferation, many researchers have focused on the role of SIRT2 in tumorigenesis, as SIRT2 is involved in cell cycle progression, cellular necrosis, and cytoskeleton reorganization^13,15^. Whether SIRT2 is a tumor suppressor^16–19^ or oncogene^20–23^ remains controversial. Recently, it was reported that suppression of SIRT2 by miR-200c alters the acetylation levels of glycolyic enzymes, which in turn facilitates cellular reprogramming during human induced pluripotency^24^. Human iPSCs and mouse iPSCs have different characteristics, including in their metabolic strategies, as they exist in primed and naive states, respectively^25^. However, the role of SIRT2 in murine cell reprogramming toward pluripotency has not been examined.

In this study, we found that complete depletion of SIRT2 prevents the generation of pluripotent stem cells from mouse embryonic fibroblasts (MEFs). We also demonstrated the production of functionally competent naive iPSCs with self-renewal capacity that differentiated into three germinal layers both in vitro and in vivo with blastocyst chimera formation, even from SIRT2-knockout (KO) MEFs; however, reprogramming efficiency was significantly low.

## Materials and methods

### iPSC generation from MEFs

Lentiviruses encoding a doxycycline (dox)-inducible polycistronic human OCT4, Sox2, Klf4, and c-Myc cassette (TetO-FUW-OSKM, #20321, Addgene, Cambridge, UK) or reverse tetracycline transactivator (FUW-M2rtTA, #20342, addgene, Cambridge, UK) were prepared from 293FT cells. MEFs were freshly isolated from SIRT^+/+^ (WT), SIRT2^+/−^ (HT), and SIRT2^−/−^ (KO) mice (Figure S1) and seeded at 1 × 10^5^ cells per 35-mm dish 1 day before viral transduction. At day 0, OSKM lentivirus and M2rtTA lentivirus (both at a multiplicity of infection = 10) and 10 μg/mL polybrene were used to infect MEFs. Two days later, transduced MEFs were passaged onto mitotically inactivated MEF feeder cells or 0.1% gelatin-coated dishes for feeder-free reprogramming. Subsequently, high-glucose Dulbecco's Modified Eagle's Medium (DMEM) (#11965-092, Thermo Fisher Scientific, MA, USA) supplemented with 10% fetal bovine serum (#12483-020, Thermo Fisher Scientific, MA, USA) was replaced with mouse embryonic stem (ES) media supplemented with 2 μg/mL dox (D9891, Sigma Aldrich, MO, USA) and high-glucose DMEM, 15% fetal bovine serum, 1% GlutaMAX (#35050-061, Thermo Fisher Scientific, MA, USA), 1% MEM-NEAA (#11140-050, Thermo Fisher Scientific, MA, USA), 1% penicillin–streptomycin (#15140-122, Thermo Fisher Scientific, MA, USA), 1 × 10^−4^ M 2-mercaptoethanol (#0482, Amresco, OH, USA), and 10^3^ U/mL leukemia inhibitory factor (ESG1106, Merck, Darmstadt, Germany). ES media supplemented with dox was changed every other day until ES-like colonies appeared. For further analyses, iPSC colonies were picked and subcultured on feeder cells.

### Semi-quantitative and quantitative reverse transcription PCR

Total RNA was extracted from cells or tumor tissues using TRIzol reagent (#15596-026, Thermo Fisher Scientific, MA, USA). Extracted RNA was then used as a template for reverse transcription into complementary DNA (cDNA) with an M-MLV cDNA synthesis kit (#28025013, Thermo Fisher Scientific, MA, USA). Semi-quantitative RT-PCR was performed using the primers shown in Table S1. cDNA was also amplified by quantitative PCR using a Rotor-Gene Q (#9001550, Qiagen, Hilden, Germany) with a Rotor-Gene SYBR Green PCR kit (#204174, Qiagen, Hilden, Germany); primers used are listed in Table S2. The results were evaluated by Rotor-Gene Q series software. The threshold cycle was determined and the relative gene expression ratio was calculated as follows, fold-change = 2 ^ΔΔCt^. Experiments were performed in triplicate.

### FACS analysis

SIRT2-WT/HT/KO-MEFs in the middle stage of reprogramming (SIRT2-WT/HT/KO-RPG) at day 18, normal MEFs as a negative control, and ESCs as a positive control were subjected to fluorescence-activated cell sorting (FACS) analysis. Fluorescein isothiocyanate (FITC) anti-mouse CD44 antibodies (#553133, BD Biosciences, NJ, USA) as a MEF marker and Alexa Fluor 647 anti-mouse SSEA-1 antibodies (#125607, BioLegend, CA, USA) or OCT3/4^−^Alexa Fluor 647 (#653710, BioLegend, CA, USA) as pluripotent stem cell markers were simultaneously used for staining. Analysis was performed by flow cytometry (FACSAria III; BD Biosciences, NJ, USA). FITC-conjugated mouse immunoglobulin Gs (#555748, BD Biosciences, NJ, USA) and Alexa Fluor 647-conjugated mouse immunoglobulin Gs (#400130, BioLegend, CA, USA) were used as negative controls during staining. Experiments were performed in triplicate.

### Differentiation of iPSCs

Both SIRT2-WT-iPSCs and SIRT2-KO-iPSCs were randomly differentiated into three germ layers through the formation of embryoid bodies using a suspension culture method on bacterial culture dishes, as described previously^26^.

### Immunofluorescence

Cells were plated and cultured on 0.1% gelatin (CA087-100, GenDepot, TX, USA)-coated coverslips. Paraformaldehyde (4%) was used to fix the cells for 15 min at room temperature. After thorough washing with phosphate-buffered saline (#10010-023, Thermo Fisher Scientific, MA, USA), cells were then permeabilized with ice-cold methanol for 10 min at − 20 °C. Blocking was performed with 3% bovine serum albumin (A2153, Sigma Aldrich, MO, USA) for 1 h. Primary antibodies used in this study were as follows: OCT3/4 (1:200; sc-5279, Santa Cruz Biotechnology, TX, USA), SIRT2 (1:100; S8447, Sigma-Aldrich, MO, USA), β-III-tubulin (1:1000; ab41489, Abcam, Cambridge, UK), α-smooth muscle actin (1:200; A5228, Sigma Aldrich), and SOX17 (1:50; ab191699,Abcam, Cambridge, UK). As secondary antibodies, anti-chicken Alexa Fluor 647 (ab150171, Abcam, Cambridge, UK), anti-mouse FITC (ab6785, Abcam, Cambridge, UK), and anti-rabbit TRITC (ab6718, Abcam, Cambridge, UK) were used at a dilution of 1:500. Nuclear counterstaining was performed with DAPI (D9542, Sigma Aldrich, MO, USA) for 10 min.

### Teratoma formation and histopathology

Six-week-old male Balb/c nu/nu mice (Orient Bio, Gyeong-gi, South Korea) were used in this assay. Animal experiments were performed in accordance with the NIH Guide for the Care and Use of Laboratory Animals and approved by the Institutional Animal Care and Use Committee of Kyungpook National University (KNU 2014^−^0167). SIRT2-WT/KO-iPSCs (5 × 10^6^) were counted and mixed with Matrigel (#8482, BD Biosciences, NJ, USA). The mixture was then injected subcutaneously into the dorsal part of nude mice. After 3 weeks, mice were killed and tumor tissues from the injected sites were isolated for histopathological and molecular analyses. Half of the tumor tissues were frozen for molecular studies and the rest were fixed in 10% neutral-buffered formalin, processed using routine methods, and embedded in paraffin wax. The samples were cut into 4-μm thick sections and then deparaffinized in toluene and rehydrated in a graded alcohol series. The sections were then stained with hematoxylin and eosin.

### Chimera formation analysis

iPSCs were electroporated with pCXLE-EGFP (#27082, Addgene, Cambridge, UK) to detect their contribution in chimeric embryos. GFP^+^ iPSCs were aggregated with denuded post-compaction eight-cell-stage embryos to obtain an aggregate chimera. Eight-cell embryos flushed from 2.5 days post coitum (dpc) B6D2F1 female mice (Charles River, Cambridge, UK) were cultured in microdrops of embryo culture medium. After the cells were trypsinized, clumps of iPSCs were selected and transferred into microdrops containing zona-free eight-cell embryos. Morula-stage embryos aggregated with iPSCs were cultured overnight at 37 °C in 5% CO_2_. The aggregated blastocysts were then transferred into the uterine horns of 2.5 dpc pseudopregnant recipients.

### Cell cycle analysis

SIRT2-WT/KO-MEFs were incubated at 100% confluency for 72 h without addition of fresh medium for cell cycle synchronization following a protocol described in a previous study^27^. Synchronized cells were then subcultured to restart the cell cycle and then harvested for propidium iodide staining after 24 h of culture. Cells (1 × 10^6^) were fixed in cold 70% ethanol for 30 min, washed with phosphate-buffered saline, treated with 100 μg/mL of RNase A (R1253, Thermo Fisher Scientific, MA, USA), and then stained with 400 μL propidium iodide solution (P4864, Sigma Aldrich, MO, USA) at room temperature overnight. Analysis was performed by flow cytometry using a FACSAria III. Experiments were performed in triplicate.

### Immunoblot analysis

Total cell lysates of SIRT2-WT/KO-MEFs were used for protein preparation. Proteins were separated on 10–15% sodium dodecyl sulphate–polyacrylamide gels and transferred to polyvinylidene fluoride membranes. Blocking was performed with 5% skim milk in Tris-buffered saline with Tween 20 (TBS/T) for 1 h. Primary antibodies used were rabbit anti-GAPDH (1:2000; #2118, Cell Signaling Technology, MA, USA), rabbit anti-SIRT2 (1:1000; S8447, Sigma-Aldrich, MO, USA), and mouse anti-p16 (1:1000; sc-166760, Santa Cruz Biotechnology, TX, USA). Horseradish peroxidase-conjugated anti-rabbit IgG (1:10,000; #7074, Cell Signaling Technology, MA, USA) or anti-mouse IgG (1:10,000; #7076, Cell Signaling Technology, MA, USA) were used as secondary antibodies. Specific binding was detected using the SuperSignal West Dura Extended Duration Substrate (#34075, Thermo Fisher Scientific, MA, USA) followed by exposure to medical X-ray film (Agfa, Mortsel, Belgium).

### Proteomics

Proteomic analyses were conducted using SIRT2-WT/KO-MEFs. In brief, 44 paired spots that showed differential expression were selected from 2D gel electrophoresis (2-DE) gels. Spots of interest were digested by trypsin and subjected to liquid chromatography-tandem mass spectrometry analysis. Peptides were searched based on the Mascot algorithm (Matrix Science, MA, USA) and filtered with a significance threshold of *P* < 0.05. Details are presented in the supporting information. Among the proteins identified with > 95% confidence and having two or more matched peptides, the rank 1 protein of each spot, except for unnamed proteins, was included in the final data analysis. Categorization of proteins and pathway analysis were performed using the DAVID program.

### Statistical analysis

All values are presented as the mean ± standard error of the mean. Statistical analyses were performed by Student’s *t* test or one-way analysis of variance followed by Bonferroni or Tukey’s honest significant difference post hoc tests for multiple comparisons. Statistical significance values were defined as *P < 0.05 or **P < 0.01.

## Results

### SIRT2 expression depends on cell potency

**Fig. 1:**
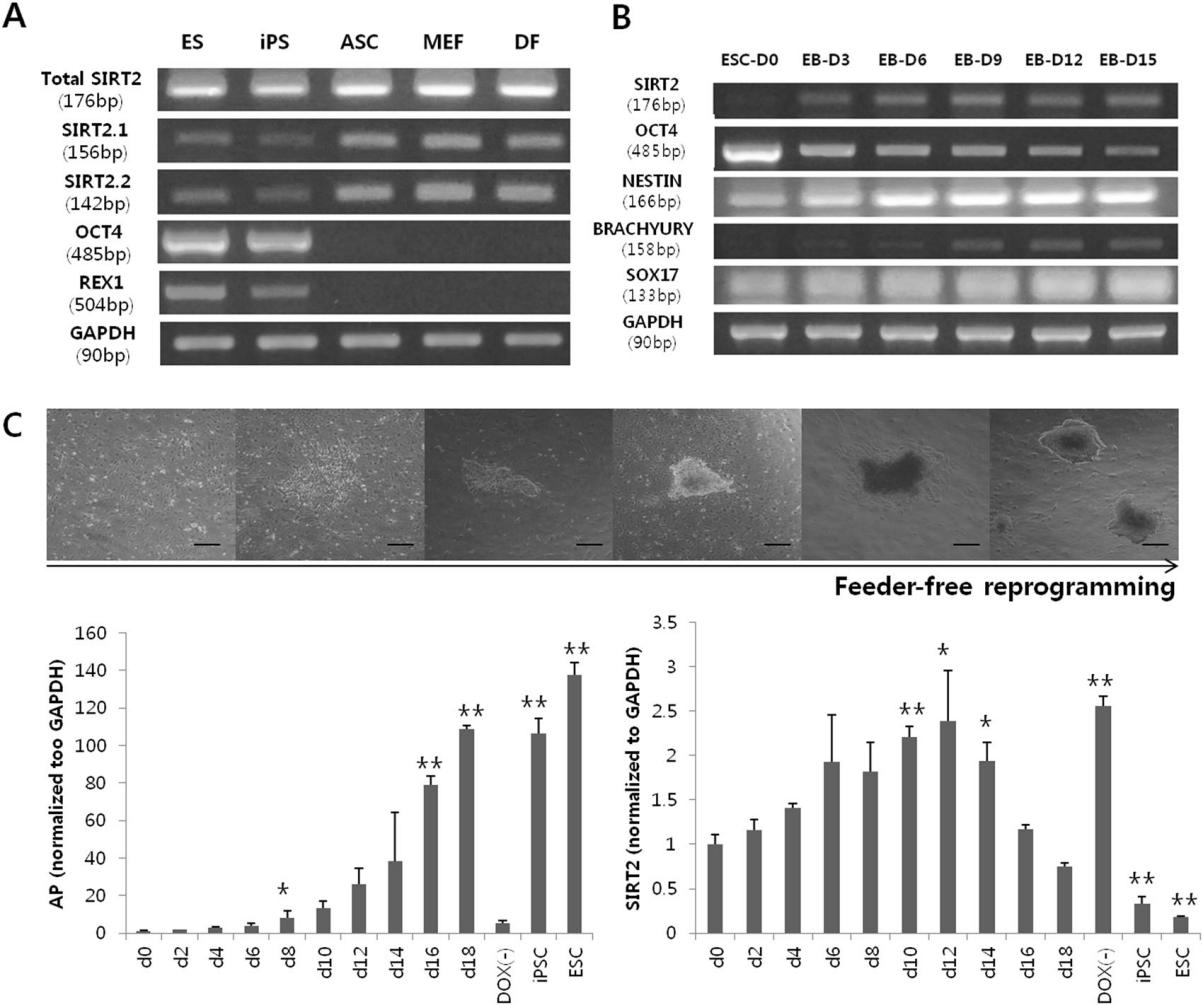
SIRT2 expression in various cell stages. **a** Expression of SIRT2 variant 1 (SIRT2.1), SIRT2 variant 2 (SIRT2.2), and total SIRT2 mRNA is dependent on cell potency. **b** Expression levels of SIRT2 during differentiation of ES cells assessed by embryoid body (EB) formation. **c** Morphological changes in cells during feeder-free reprogramming. Scale bar = 400 μm. Expression of alkaline phosphatase (AP) is shown as a positive control. mRNA expression levels of SIRT2 slightly increased in the middle stage of reprogramming, but drastically decreased as reprogramming was completed. *P *<* 0.05 and **P *<* 0.01 compared with d0 (*n* = 3)

SIRT2 was differentially expressed in several cell types depending on their potency. We examined various murine cells, including pluripotent stem cells such as embryonic stem cells and iPSCs, adipose-derived stem cells as multipotent stem cells, and unipotent somatic cells including MEFs and dermal fibroblasts. Expression of SIRT2 showed an inverse correlation with the pluripotency marker OCT4 and naive state marker REX1. Unipotent somatic cells exhibited the most robust SIRT2 expression, whereas pluripotent cells showed weak expression (Fig. 1a). A similar tendency was observed during the spontaneous differentiation of embryonic stem cells (Fig. 1b). SIRT2 expression increased as differentiation progressed; however, a slight decrease was observed by day 9 along with further differentiation. This was not a lineage-specific phenomenon, as the same expression pattern was observed during retinoic acid-induced embryonic stem cell differentiation (data not shown). We also examined whether the expression of SIRT2 continuously decreased during pluripotency reprogramming using a feeder-free reprogramming strategy. Unexpectedly, SIRT2 increased before the mid-stage of reprogramming, after which a sudden decrease was observed (Fig. 1c).

### SIRT2 deficiency inhibits cellular reprogramming to naive pluripotent stem cells

**Fig. 2:**
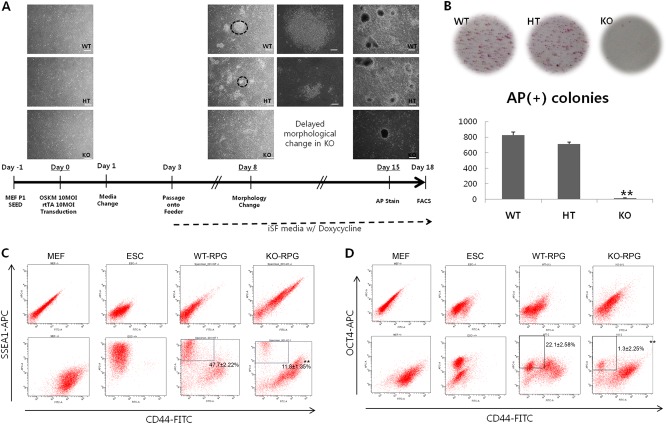
Low efficiency and slow kinetics during reprogramming of SIRT2-knockout cells. **a** Delayed morphological changes in SIRT2-KO-MEFs. Scale bar = 400 μm. **b** Number of alkaline phosphatase-positive colonies. **c** Proportion of the SSEA-1(+)/CD44(−) fraction. **d** Proportion of the OCT4(+)/CD44(−) fraction. Non-reprogrammed MEFs are shown as negative control and mouse ESCs are shown as positive control. Samples treated with FITC- and Alexa Fluor 647-conjugated mouse immunoglobulin Gs (IgGs) are presented in the upper panel as negative controls for staining. *P < 0.05 and **P *<* 0.01 compared with wild-type (*n* = 3). See also figure S1

SIRT2-KO-MEFs showed delayed progression in reprogramming. Mesenchymal–epithelial transition was not observed in KO cells by day 8 of reprogramming and most colonies formed by KO cells on day 15 were non-ES-like, exhibiting a flat shape and blunt border (Fig. 2a). Alkaline phosphatase (AP) staining revealed only a few positive colonies of KO cells; however, there was no distinct difference between wild-type (WT) cells and hetero-type (HT) cells (Fig. 2b). Thus, further experiments without HT cells were performed to compare WT and KO cells. Based on FACS analysis, the CD44^−^/SSEA-1^+^ and CD44^−^/OCT4^+^ fractions indicative of cells in early or late stages of reprogramming, respectively, were also significantly diminished in KO cells compared with in WT cells (Fig. 2c, d).

### SIRT2-KO-iPSCs are in a state of naive pluripotency

**Fig. 3:**
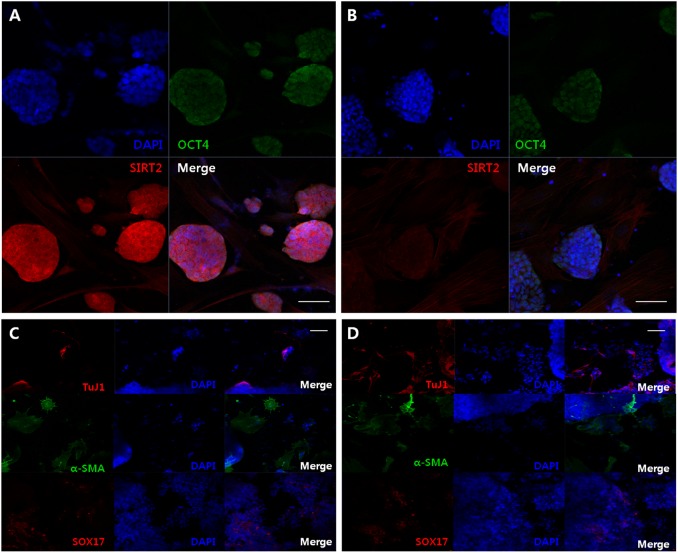
Characterization of SIRT2 wild-type and knockout iPSCs in vitro. Immunofluorescence of the pluripotency marker OCT4 in **a** WT-iPSCs and **b** KO-iPSCs. Scale bar = 50 μm. Immunofluorescence of the three germ layer differentiation markers in differentiated **c** WT-iPSCs and **d** KO-iPSCs assessed by embryoid body formation. Tuj1 for ectoderm, α-SMA for mesoderm, and SOX17 for endoderm. Scale bar = 100 μm. Representative images are from experiments performed in triplicate

SIRT2-KO cells showed decreased ES-like iPSC colony formation compared with their WT counterparts. Thus, it was unknown whether SIRT2-KO-iPSCs were functional pluripotent stem cells. To address this, both SIRT2-WT-iPSCs and SIRT2-KO-iPSCs were simultaneously characterized and compared. iPSC lines were established through picking and propagation of independent ES-like colonies. Finally, five pairs of SIRT2-WT-iPSC and SIRT2-KO-iPSC lines were selected, as these clones were highly proliferative and could be passaged for long periods of time without differentiation. When these undifferentiated cells were subjected to immunofluorescence, both iPSC lines expressed the pluripotent stem cell marker OCT4 in the nucleus, irrespective of SIRT2 expression in the cytoplasm (Fig. 3a, b). Furthermore, these cell lines differentiated into tissues from all three germinal layers upon differentiation through embryoid body formation in vitro (Fig. 3c, 3d).

**Fig. 4:**
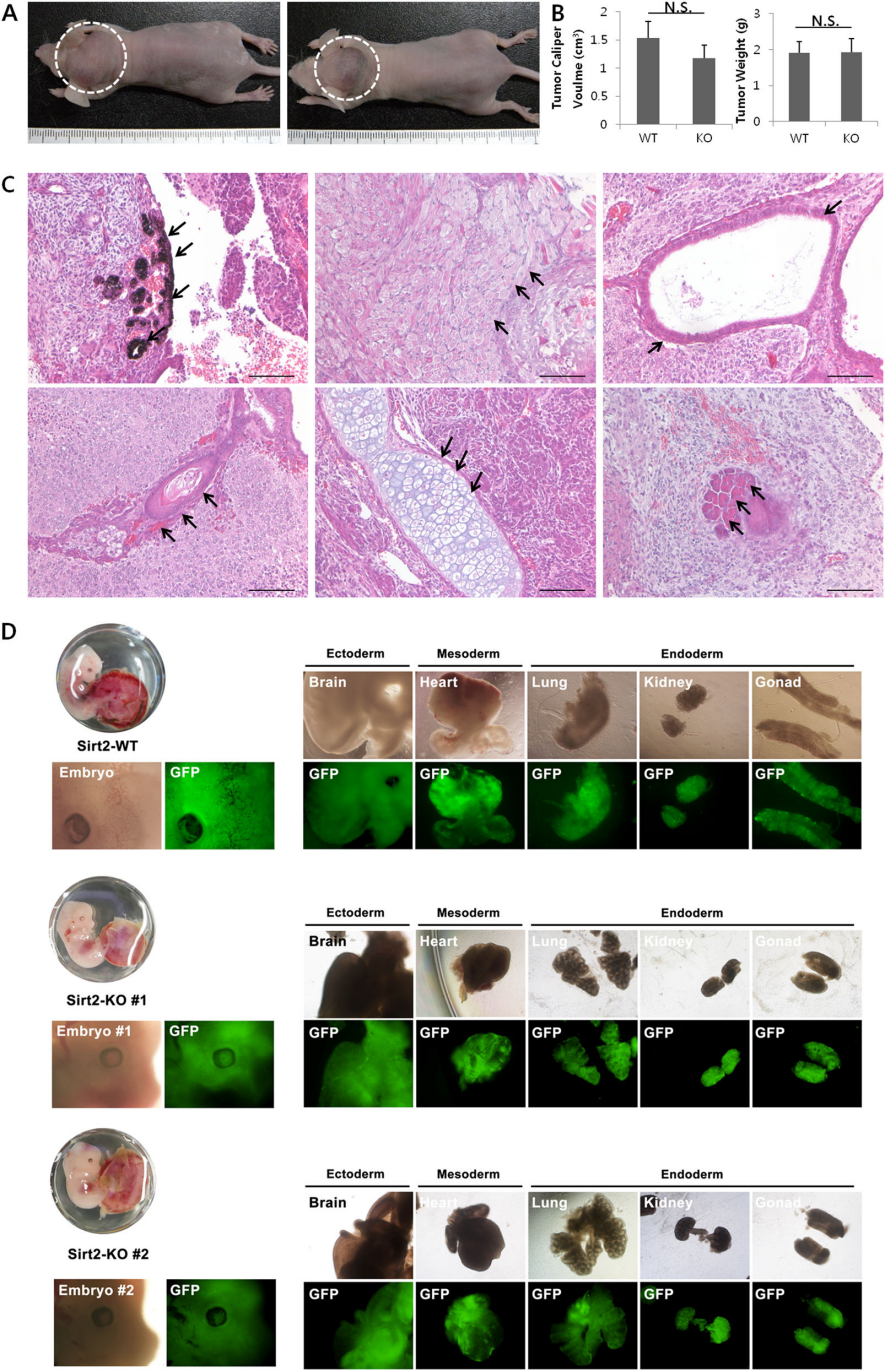
In vivo developmental potential of iPSCs. **a** WT (left) and KO (right) iPSCs formed tumors in immunodeficient mice. Representative images are from five mice per group. **b** Tumor tissues from both groups showed no significant differences in size and weight. **c** Tumor tissues from both groups were diagnosed as teratomas. A WT-iPSC-derived teratoma is shown in the upper row and a KO-iPSC-derived teratoma is shown in the bottom row. From left, ectoderm, mesoderm, and endoderm tissues are indicated by arrows. Scale bars = 200 μm. *P < 0.05, **P < 0.01 compared with wild-type (*n* = 5). **d** Chimera formation by aggregation with SIRT2-WT-iPSCs (upper panel) or SIRT2-KO-iPSCs (middle and lower panel). Embryos (13.5 dpc) showed that GFP^+^ cells (SIRT2-WT-iPSCs and SIRT2-KO-iPSCs) contribute to all three germ layers of ectoderm, mesoderm, and endoderm as well as gonads

Moreover, SIRT2-KO-iPSCs and SIRT2-WT-iPSCs formed tumors upon injection into immunodeficient mice (Fig. 4a). The size and weight of tumor tissues derived from SIRT2-WT-iPSCs and SIRT2-KO-iPSCs were similar (Fig. 4b). Based on histopathology, the tumors were diagnosed as teratomas, which contain derivatives of all three germ layers. Pigment cells or epidermis were identified as ectoderm-derived components, skeletal muscle, and cartilage as mesoderm-derived components, and respiratory epithelia or acinar cells as endoderm-derived components (Fig. 4c). Thus, both iPSC lines are functional pluripotent stem cells, as they could differentiate into all three germ layers in vivo and in vitro.

To validate whether SIRT2-KO-iPSCs are in a naive pluripotent state, we tested for chimera formation. Both SIRT2-WT and SIRT2-KO-iPSCs contributed to the formation of three germinal layers and gonads of chimeras (Fig. 4d), although SIRT2-KO-iPSC-derived chimeras showed frequent embryonic lethality during 10.5–11.5 days post coitum (Figure S2). The methylation status of OCT4 and Nanog promoter regions in SIRT2-WT-iPSC, SIRT2-KO-iPSC, SIRT2-WT-MEF, and SIRT2-KO-MEF cell lines were also analyzed by quantitative pyrosequencing. For the OCT4 gene, both SIRT2-MEF cell lines (SIRT2-WT-MEF, 75.9%; SIRT2-KO-MEF, 74.6%) showed a significantly higher methylation status compared with in the SIRT2-iPSC lines (SIRT2-WT-iPSC, 5.4%; SIRT2-KO-iPSC, 4.5%). However, there was no difference in OCT4 gene methylation between SIRT2-WT and SIRT2-KO cell lines (Figure S3A). Nanog gene methylation showed a similar trend to that of OCT4 (Figure S3B).

### SIRT2 depletion impairs cell cycle progression

**Fig. 5:**
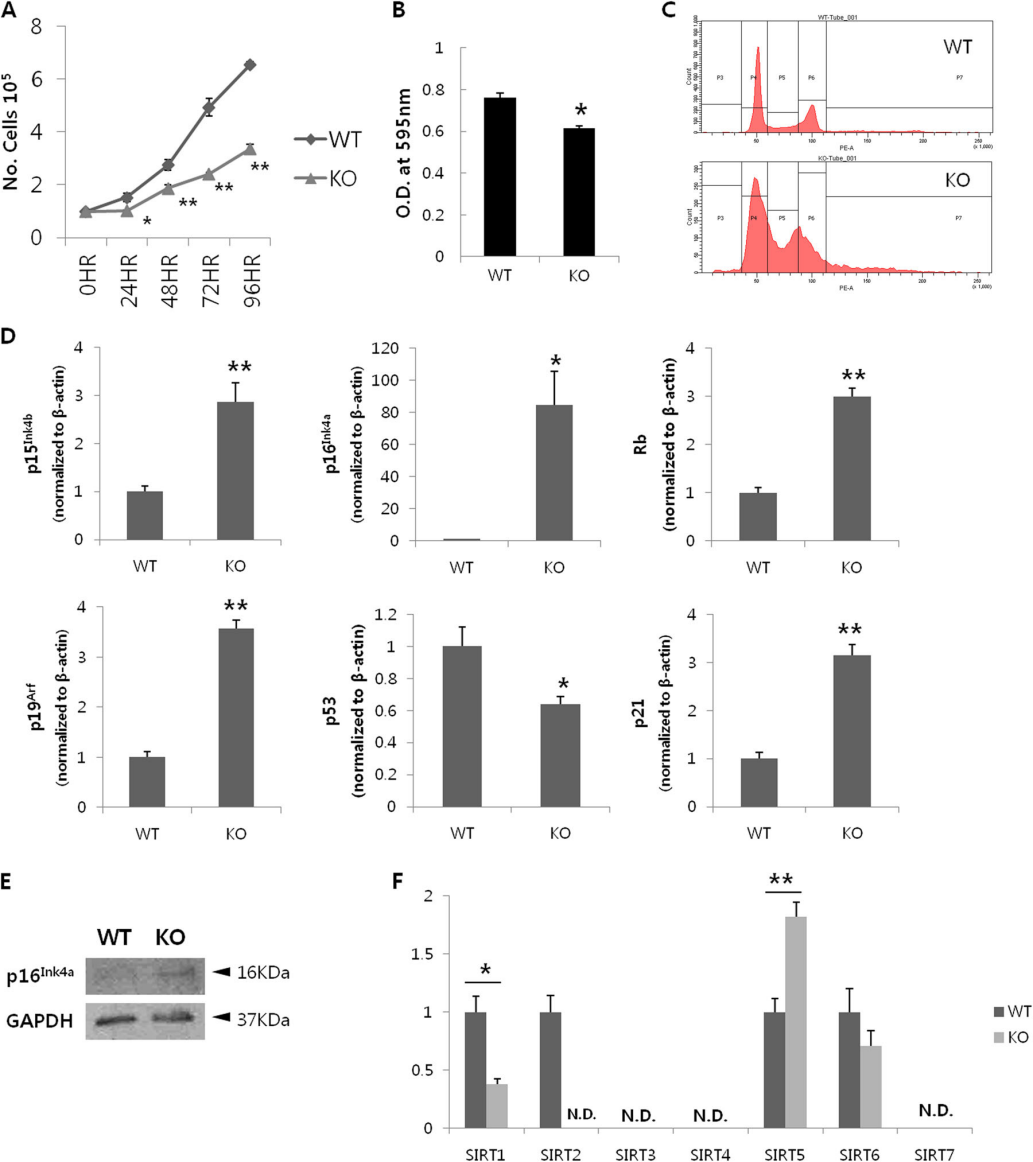
SIRT2 depletion results in aberrant cell cycle progression. **a** Cell proliferation rate assessed and compared by manual counting. **b** Cell viability assessed and compared based on WST-1 assays. **c** Cell cycle analysis was performed by propidium iodide staining after synchronization. Representative images are from triplicate experiments using three independent cell lines. **d** Relative mRNA expression levels of INK4/ARF locus components (p15^Ink4b^, p16^Ink4a^, and p19^Arf^) and their downstream effectors (Rb, p53, and p21) were assessed. **e** Western blotting of P16^Ink4a^, which showed the most prominent difference at the mRNA level. **f** Relative mRNA expression levels of all sirtuin members. N.D., not detected. *P < 0.05, **P < 0.01 compared with wild-type (*n* = 3). Original images are shown in Figure S4

Although SIRT2-KO-MEFs were hypothesized to be reprogrammed better than their WT counterparts as was observed for human cells, the results did not support this hypothesis. However, the resultant KO-iPSCs functioned similarly to wild-type iPSCs. Therefore, we focused on the differences between SIRT2-WT-MEFs and SIRT2-KO-MEFs to identify the reason for the decreased reprogramming efficiency in KO cells. During cell culture, SIRT2-KO-MEFs required more time to reach confluency. Based on manual counts (four times at 24-h intervals) and WST-1 assays, KO cells showed reduced proliferation compared to normal cells (Fig. 5a, b). Because SIRT2 is a well-known cell cycle regulator, we predicted that the difference was because of the different cell cycle distribution between SIRT2-KO-MEFs and WT MEFs. As expected, SIRT2-KO-MEFs displayed an abnormal pattern, as 2 N and 4 N peaks partially overlapped without separation by an S-phase fraction (Fig. 5c). This indicates that depletion of SIRT2 resulted in aberrant cell cycle progression, in turn causing a delay in cell growth and reprogramming. Because genes involved in both cell cycle regulation and iPSC reprogramming required further investigation, we focused on the INK4/ARF locus based on a previous study^28^. The main transcriptional products of INK4/ARF and their downstream gene expression were analyzed by quantitative PCR. p16^Ink4a^, p15^Ink4b^, Rb, p19^Arf^, and p21, but not p53, were upregulated in SIRT2-KO-MEFs compared with in WT cells. Particularly, p16^Ink4a^ was markedly differentially expressed between the cell types, showing an ~ 80-fold increase in mRNA expression levels in KO cells compared with in WT cells (Fig. 5d). The difference in p16^Ink4a^ expression between the two cell types was also confirmed at the protein level. INK4/ARF products are rarely expressed in growing primary cells; indeed, WT MEFs exhibited virtually undetectable expression. However, INK4/ARF protein expression was observed in KO cells (Fig. 5e). Moreover, all members of the SIRT family were analyzed because the deletion of SIRT2 may affect the expression of other SIRTs, some of which are known to regulate reprogramming efficiency. Lower expression levels of SIRT1 were observed in KO-MEFs, whereas SIRT5 expression was higher in KO-MEFs, showing an inverse correlation with SIRT2 expression. SIRT6 expression did not significantly differ between the two cell types (Fig. 5f). Although neither SIRT1 and SIRT5 expression levels was affected when the SIRT2 was artificially downregulated by transfection of siRNA for SIRT2 (Figure S4E), the inverse correlation between SIRT2 and SIRT5 was consistently observed when their natural expression levels were compared in between the wild-type cells and SIRT2-KO cells, including iPSCs (Figure S4F).

### Proteome profile depends on SIRT2 expression of MEFs

The greatest difference in proliferation rates was observed between wild-type MEFs and SIRT2-KO-MEFs, and thus cell cycle and related regulatory genes were analyzed. In addition, the proteomes of WT and KO cells were compared with identify other underlying causes of decreased reprogramming efficiency of SIRT2-knockout MEFs. In 2-DE gel analysis, 215 paired protein spots and 237 non-paired protein spots were detected. Among the paired spots, 25 were morphometrically increased by more than twofold in KO cells compared with in WT cells. In addition, 19 spots decreased by > 50% in KO cells compared with in WT cells. Differentially expressed spots were subjected to liquid chromatography-tandem mass spectrometry. The cutoff criteria at a *P* value < 0.05 and fold-ratio difference > 2 was strictly adjusted and the rank 1 protein for each spot satisfying the criteria is summarized in Table 1. Subsequently, differentially expressed proteins were analyzed for biological process, cellular component, and molecular function. Among the 242 affected biological processes, seven were significant at *P* < 0.05 with Bonferroni correction; all were related to conformational changes (Figure S5A). Among the 65 affected cellular components, 18 were significant at *P* < 0.05 with Bonferroni correction; the top 10 components, which contained cytoplasm or exocytosis-related compartments, are shown in Figure S5B. Among the 43 affected molecular functions, nine were significant at *P* < 0.05 with Bonferroni correction; these were all binding functions, which were further classified into various biological activities (Figure S5C). Next, pathways affected by SIRT2 depletion were analyzed using the KEGG database. The top 10 significant pathways (among 19 pathways) at *P* < 0.05 are presented in Figure S5D. Consistent with these findings, the cell cycle-related pathway was identified. Moreover, the pathways most affected by SIRT2 depletion were the PI3K-Akt signaling pathway followed by the Hippo signaling pathway, both of which are known to be closely related to iPSC reprogramming.

**Table 1:** Proteins differently expressed between SIRT2-knockout (KO) MEFs and wild-type (WT) MEFs^a^

No.	Spot. no.	Accession no.	Symbol	Description	Protein Score	Peptide no.^b^	KO/WT ratio^c^
1	52	gi34328108	Col1a1	Collagen alpha-1(I) chain precursor	908	26	0.16
2	906	gi225579033	Idh2	Isocitrate dehydrogenase (NADP), mitochondrial precursor	210	4	0.22
3	364	gi6677691	Rcn1	Reticulocalbin-1 precursor	285	11	0.23
4	762	gi6677833	S100a10	Protein S100-A10	86	3	0.27
5	72	gi19909851	Dbn1	Drebrin A	269	13	0.30
6	546	gi34328230	Ak2	Adenylate kinase 2, mitochondrial isoform b	155	4	0.38
7	458	gi3329498	Hnrnpa2b1	Heterogenous nuclear ribonucleoprotein A2/B1	294	7	0.39
8	905	gi28201978	Pdhx	Pyruvate dehydrogenase protein X component, mitochondrial	213	3	0.41
9	97	gi6755863	Hsp90b1	Endoplasmin precursor	348	15	0.41
10	329	gi6680840	Calu	Calumenin isoform 1 precursor	336	6	0.42
11	341	gi6755100	Pa2g4	Proliferation-associated protein 2G4	272	6	0.43
12	154	gi21704156	Cald1	Caldesmon 1	550	17	0.43
13	334	gi188035858	Rcn3	Reticulocalbin-3 precursor	328	5	0.47
14	109	gi6754254	Hsp90aa1	Heat shock protein HSP90-alpha	124	5	0.47
15	285	gi2078001	Vim	Vimentin	428	16	0.49
16	395	gi18079341	Eif3h	Eukaryotic translation initiation factor 3 subunit H	251	6	0.49
17	584	gi6756039	Ywhaq	14-3-3 protein theta	93	3	2.02
18	197	gi13384620	Hnrnpk	Heterogeneous nuclear ribonucleoprotein K	291	8	2.04
19	283	gi15489222	Etf1	Eukaryotic translation termination factor 1	92	2	2.04
20	927	gi2078001	Vim	Vimentin	703	25	2.05
21	916	gi8393544	Hnrnpc	Heterogeneous nuclear ribonucleoproteins C1/C2, isoform 1	250	8	2.05
22	918	gi809561	Actg1	Gamma-actin	244	8	2.19
23	328	gi31980808	Eif3g	Eukaryotic translation initiation factor 3 subunit G	193	7	2.23
24	213	gi148691054	Usp14	Ubiquitin specific peptidase 14, isoform CRA_a	179	6	2.35
25	469	gi6678131	Srm	Spermidine synthase	287	8	2.56
26	377	gi283436180	Hnrnpc	Heterogeneous nuclear ribonucleoproteins C1/C2, isoform 3	161	5	2.59
27	340	gi27370092	Tufm	Elongation factor Tu, mitochondrial, isoform 1	242	8	2.66
28	134	gi29568084	Snx9	Sorting nexin-9	169	3	2.80
29	907	gi6754994	Pcbp1	Poly(rC)-binding protein 1	162	4	2.88
30	896	gi6753494	Coro1b	Coronin-1B	103	5	2.90
31	534	gi3065925	Ywhab	14-3-3 protein beta	420	6	2.94
32	105	gi23271416	P3h1	Leprecan1	389	13	3.01
33	542	gi6679299	Phb	Prohibitin	293	8	3.08
34	545	gi1526539	Ywhaz	14-3-3 zeta	283	7	3.12
35	198	gi13384620	Hnrnpk	Heterogeneous nuclear ribonucleoprotein K	477	12	3.50
36	266	gi7271905	Wdr12	Nuclear protein Ytm1p	97	3	3.78
37	909	gi192659	Acads	Short chain acyl-CoA dehydrogenase	289	7	4.32
38	631	gi885932	Prdx2	Peroxidase	149	6	4.50
39	227	gi51455	Hspd1	Heat shock protein 65	200	6	7.52
40	201	gi13384620	Hnrnpk	Heterogeneous nuclear ribonucleoprotein K	104	3	8.79
41	372	gi21704096	Tardbp	TAR DNA-binding protein 43, isoform 1	172	4	15.74

^a^For proteomic analysis, we performed quantification based on biological triplicate experiments. Among 44 spots detected by LC-MS/MS, unnamed rank 1 proteins of three spots were excluded from further analysis. See also Figure S2.

^b^Peptides with ion scores above the 95% confidence level were counted.

^c^KO/WT ratio represents the ratio of proteins detected in SIRT2-knockout MEFs to those of wild-type MEFs. Increased expression, fold-change ≥ 2; decreased expression, fold-change ≤ 0.5.

## Discussion

Because iPSCs were first generated using Yamanaka factors with an efficiency of ~ 0.1%, numerous studies have been conducted to improve reprogramming efficiency. Typically, introduction of genes that are highly expressed in ESCs, such as Nanog and Sall4, provokes more efficient pluripotency reprogramming^29,30^. Similarly, artificial inhibition of genes that show low expression in ESCs, such as Mbd3 and p53, typically augments reprogramming^31,32^. Because SIRT2 displayed low expression levels in ESCs, its inhibition was expected to increase reprogramming efficiency, as reported for human cell reprogramming toward pluripotency^24^.

However, we demonstrated that complete depletion of SIRT2 paradoxically decreased reprogramming efficiency in murine cells. This result may be attributed to the high transient expression of SIRT2 during the mid-stage of reprogramming, rather than a gradual decrease from day 0 to the end of iPSC reprogramming (Fig. 1c). The epithelial–mesenchymal transition factor SNAIL paradoxically enhances reprogramming efficiency^33^; similarly, stage-specific elevation of SIRT2 appears to be required for successful reprogramming. After SIRT2 depletion, the kinetics of reprogramming and resultant iPSC colonies were decreased compared with those of WT or HT cells. Based on a previous study^34^ that used the same inducible lentiviral system for reprogramming as this study, AP was found as the most initially activated pluripotency marker and subsequent activation of SSEA-1 represented a transgene-dependent partially reprogrammed stage. When the MEFs were fully reprogrammed and independent from the transgenes, OCT4 was expressed^34^. We found that initial activation of AP was most severely inhibited in SIRT2-KO-MEFs during the initial stage of reprogramming, although sequential activation of SSEA-1 and OCT4 were also depressed under SIRT2-KO conditions (Fig. 2). Because SIRT2 expression gradually increased from the initial stage to the mid-stage of reprogramming, SIRT2 may play a positive role in AP activation during this period and before activation of SSEA-1.

As the induction and maintenance of pluripotency both require activation of similar gene sets, we examined whether SIRT2-KO-iPSCs are fully functional iPSCs. Although depletion of SIRT2 prevents the reprogramming process, established ES-like iPSC lines from SIRT2-KO cells showed characteristic features of pluripotent stem cells, such as a large N/C ratio, rapid proliferation, and OCT4 expression, when cultured on feeder cells with ES media. This result is also consistent with that of a previous study, which demonstrated that SIRT2 knockdown did not affect OCT4 and Nanog expression in mouse ESCs^35^. When cultured without leukemia inhibitory factor, SIRT2-KO-iPSCs spontaneously formed embryoid bodies in non-adherent conditions and were further differentiated into the ectodermal, mesodermal, and endodermal linages. The functionality of these cells was also demonstrated in vivo by teratoma and chimera formation. Although significantly reduced in number, SIRT2-KO-iPSCs retained pluripotent competency, indicating that there is functional redundancy of SIRT2. There is evidence that not only SIRT2 but also SIRT1 possess H4K16Ac deacetylase activity; this suggests a synergistic relationship or functional redundancy between SIRT1 and SIRT2^36^. Because SIRT1 also plays a positive role during reprogramming^9^, it may be a functional substitute for SIRT2 in SIRT2-KO cells.

To further explore this phase-specific effect mediated by SIRT2 depletion during reprogramming, we compared WT MEFs and SIRT2-KO-MEFs. Although many previous studies of SIRT2 have used immortalized cells for research^17,37,38^, immortalized cells may contain malfunctioning tumor suppressor genes. Although immortalized MEFs generated by the classic 3T3 protocol are known to possess functional p53^39^, these cells are not identical to primary MEFs, as they were shown to be resistant to miR-290 senescence cues, irrespective of p53 status^40^. In addition, the miR-290 family is known to be expressed abundantly in mouse embryonic stem cells and aid in reprogramming of somatic cells toward pluripotency^41^. Therefore, immortalized MEFs may not reproduce normal cell characteristics, particularly in the field of iPSC research. Thus, primary MEFs between passage numbers 1 and 5 were used in the present study.

SIRT2 is known for its cell cycle regulatory function. Previous studies showed that SIRT2 is upregulated during mitosis and that its overexpression prolongs mitosis^42^ and shortens the G1 phase^43^. Another study using primary MEFs derived from SIRT2-knockout mice reported longer G1 and shorter S phases, with rarely observed effects on mitosis duration^12^. Kim et al.^17^ recently reported that SIRT2 controls mitotic exit by regulating the anaphase-promoting complex and cyclosome activity. We also confirmed that SIRT2-knockout MEFs displayed aberrant cell cycle progression and thus considered the correlation between cell cycle and iPSC generation. The INK4/ARF tumor suppressor locus is known not only for its role in cell cycle control^44^ but also as a barrier for reprogramming^28^. p16^Ink4a^ and p15^Ink4b^ bind to and inhibit the cyclin D-dependent kinases Cdk4 and Cdk6 to subsequently relieve the cell cycle inhibitory effects of Rb, whereas p19^Arf^ binds to and inhibits Mdm2 to stabilize the tumor suppressor p53^28^. Thus, genes in the INK4/ARF tumor suppressor locus are not activated under normal physiological conditions in young animals, but show increased expression during aging to mitigate the threats of tumorigenesis by activating both Rb and p53. If Rb and p53 become inactivated, cells gain abnormally enhanced proliferative ability^45^. Meanwhile, the INK4/ARF locus is actively silenced in stem cells, such as embryonic and adult stem cells, to maintain their characteristic proliferative ability^45^. During reprogramming, exogenous transcription factors such as Klf4 and c-Myc can activate INK4/ARF-associated pathways, as they are well-known oncogenes^46,47^. Thus, the INK4/ARF locus should be silenced to facilitate reprogramming, although factors used for reprogramming ultimately activate that locus. In this regard, previous studies have revealed that inhibition of each component of the INK4/ARF-associated pathway not only enhances reprogramming efficiency, but also makes reprogramming possible with fewer transcription factors^28,32,48,49^. We observed that p16^Ink4a^, p15^Ink4b^, and p19^Arf^, three genes encoded by the INK4/ARK locus, and their respective downstream effectors, Rb and p21, were upregulated in SIRT2-KO-MEFs. Although p53, a mediator of p19^Arf^ and p21, showed slightly higher expression levels in WT cells than in KO cells, a previous study using the specific SIRT2 inhibitor tenovin-D3 revealed that SIRT2 inhibition may directly upregulate p21, irrespective of p53^50^. Another study revealed that SIRT2 inhibition by resveratrol treatment or SIRT2 knockdown using siRNA for SIRT2 in human fibroblasts induced INK4/ARF locus gene expression through DNA damage^51^. Therefore, it was theorized that SIRT2-KO-MEFs already possess higher basal expression levels of INK4/ARF components and that reprogramming-induced stress may potentiate the expression of these tumor suppressor genes. Consequently, reprogramming signals must overcome the highly expressed INK4/ARF locus in knockout cells to generate iPSCs.

Because p16^Ink4a^ is rarely expressed in normal primary cells, as shown by our results (Fig. 5e), and the absence of INK4/ARF products in proteomics may be because 2-DE spots from one of the three WT MEF protein samples were set as the standard and only paired spots were used for mass spectrometry. Among the 41 differentially expressed proteins, isocitrate dehydrogenase 2 mitochondrial precursor (IDH2), heat shock protein 90-alpha, endoplasmin precursor, 14-3-3 protein beta, 14-3-3 protein theta, and 14-3-3 protein zeta expression patterns strongly support other data in this study. IDH2 was more abundant in WT MEFs than in KO and is responsible for the production of NADPH, whereas NADPH is essential for the proliferation of both normal and tumor cells^52^. In addition, NADPH is needed to establish cancer-like glycolytic phenotypes, which is further required for establishing and maintaining a DNA methylation pattern in iPSCs resembling that of ESCs^53^. Recently, it was reported that primed human pluripotent stem cells require SIRT2 downregulation to maintain pluripotency via altering metabolic pathways toward aerobic glycolysis^24^. In contrast, naive state mouse ESCs are bivalent in their energy production, as they can switch from glycolysis to oxidative phosphorylation on demand^54^. Thus, different metabolic states between human and mouse cells may provoke different responses upon SIRT2 depletion. HSP90 has two cytosolic isoforms, HSP90α and HSP90β, and is an important stress protein^55^; both isoforms were upregulated in WT cells and are known to be crucial for maintaining pluripotency in mouse ESCs by regulating OCT4 and Nanog^56^. In contrast, 14-3-3 protein isoforms were upregulated in SIRT2-knockout cells; these proteins bind to phosphorylated TRIM32 and induce an increase in soluble free TRIM32^57^, which is known to repress iPSC reprogramming by modulating OCT4 stability^58^.

Based on gene ontology and KEGG pathway analyses, differentially expressed proteins between WT MEFs and SIRT2-KO-MEFs are important for conformational changes and binding processes. Furthermore, many of these proteins were found to be associated with the PI3K-Akt signaling pathway, which is essential for iPSC survival^59^, and Hippo signaling pathway, which acts a barrier to iPSC reprogramming^60^.

In addition, SIRT1 and SIRT5 showed altered expression in SIRT2-depleted MEFs. SIRT1, previously shown to enhance reprogramming^9^, exhibited lower expression levels in SIRT2-KO-MEFs; this may be an additional reason for the poor reprogramming efficiency of these cells. However, it is unlikely that the expression level of SIRT2 directly affects to that of SIRT1 because SIRT1 expression does not changed in case of SIRT2 knockdown by siRNA transfection. Meanwhile, the expression pattern of SIRT5 contrasted that of SIRT2, but the implication of this phenomenon is unknown as the exact association of SIRT5 with stem cells or tumorigenesis remains unclear.

**Fig. 6:**
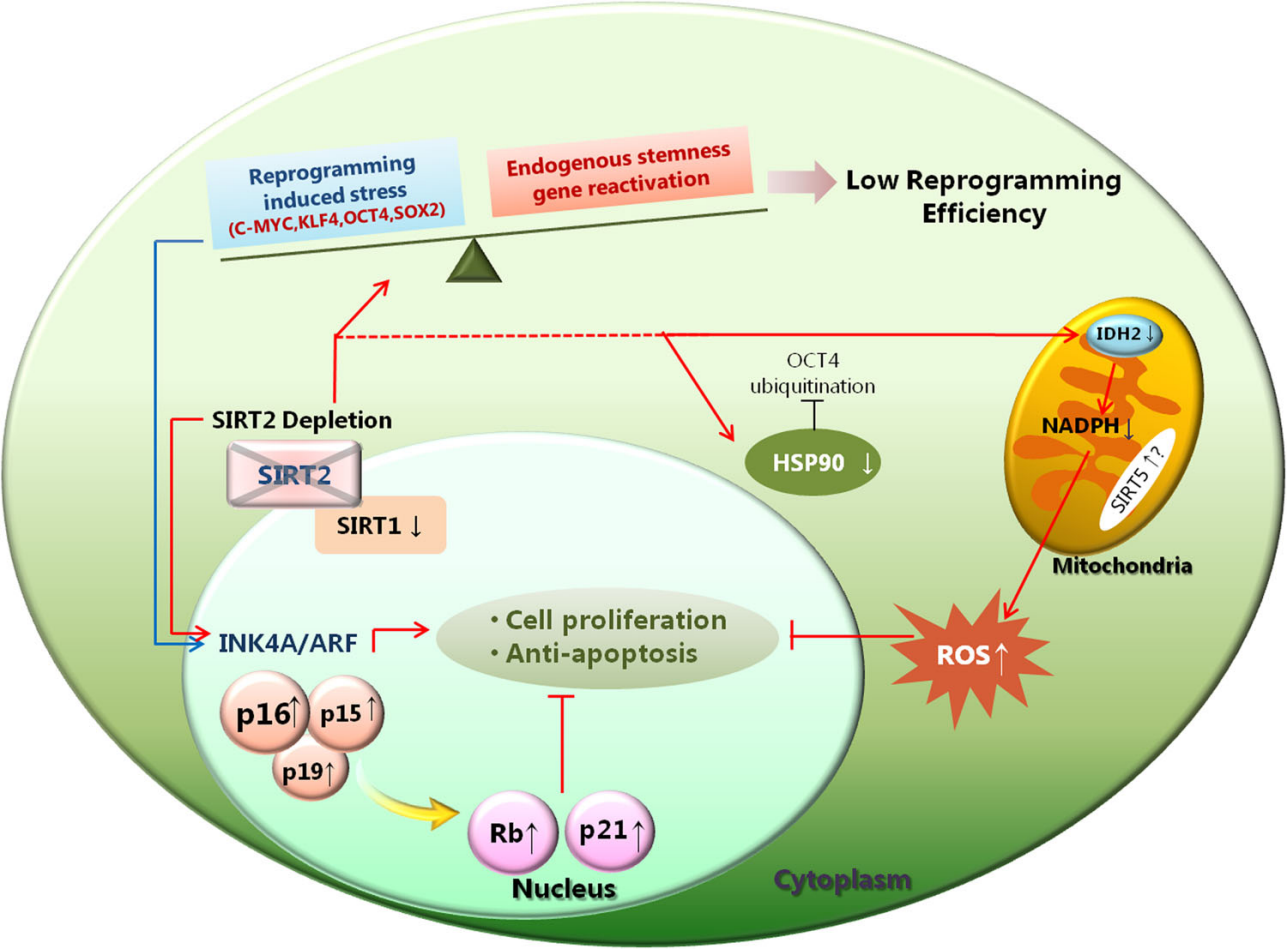
Mechanism of SIRT2 depletion effects during the reprogramming process. When reprogramming factors are introduced to mouse embryonic fibroblasts (MEFs), they provoke reprogramming-induced stress. To acquire pluripotency, somatic cells must overcome this stress and reactivate stemness genes endogenously; to achieve this, robust proliferation is a prerequisite. However, when SIRT2 is depleted, reprogramming-induced stress is apparently augmented. SIRT2-knockout MEFs show increased activated INK4/ARF signals, predominantly for p16^Ink4a^. Moreover, increased 14-3-3 protein expression may further repress stable expression of OCT4. Decreased expression of HSP90 and IDH2 negatively affects reprogramming through antiproliferation. These events may collectively limit the efficient reprogramming of SIRT2-knockout MEFs to induced pluripotent stem cells (iPSCs)

The present study provides important information regarding SIRT2-mediated alteration of gene sets required for successful reprogramming towards pluripotency. SIRT2 itself is dispensable for reprogramming towards naive pluripotency and subsequent differentiation of murine cells. However, its depletion exacerbates reprogramming-induced stress through senescence marker activation and significantly reduces reprogramming efficiency. Although the role of SIRT2 in pluripotent stem cells during phase change from naive to primed states requires further analysis, SIRT2 may assist in the efficient reprogramming of murine somatic cells to naive pluripotency (Fig. 6).

## Electronic supplementary material

Supplemental informations

## References

[1] Nakagawa T, Guarente L (2011). Sirtuins at a glance. J. Cell Sci..

[2] Bosch-Presegue L, Vaquero A (2014). Sirtuins in stress response: guardians of the genome. Oncogene.

[3] Michishita E, Park JY, Burneskis JM, Barrett JC, Horikawa I (2005). Evolutionarily conserved and nonconserved cellular localizations and functions of human SIRT proteins. Mol. Biol. Cell.

[4] Sebastian C, Satterstrom FK, Haigis MC, Mostoslavsky R (2012). From sirtuin biology to human diseases: an update. J. Biol. Chem..

[5] Knoepfler PS (2009). Deconstructing stem cell tumorigenicity: a roadmap to safe regenerative medicine. Stem Cells.

[6] Krizhanovsky V, Lowe SW (2009). Stem cells: the promises and perils of p53. Nature.

[7] Riggs JW (2013). Induced pluripotency and oncogenic transformation are related processes. Stem. Cells Dev..

[8] De Bonis ML, Ortega S, Blasco MA (2014). SIRT1 is necessary for proficient telomere elongation and genomic stability of induced pluripotent stem cells. Stem Cell Rep..

[9] Lee YL (2012). Sirtuin 1 facilitates generation of induced pluripotent stem cells from mouse embryonic fibroblasts through the miR-34a and p53 pathways. PLoS ONE.

[10] Sharma A (2013). The role of SIRT6 protein in aging and reprogramming of human induced pluripotent stem cells. J. Biol. Chem..

[11] Staszkiewicz J (2013). Silencing histone deacetylase-specific isoforms enhances expression of pluripotency genes in bovine fibroblasts. Cell Reprogram..

[12] Vaquero A (2006). SirT2 is a histone deacetylase with preference for histone H4 Lys 16 during mitosis. Genes Dev..

[13] Inoue T (2007). SIRT2, a tubulin deacetylase, acts to block the entry to chromosome condensation in response to mitotic stress. Oncogene.

[14] North BJ, Verdin E (2007). Mitotic regulation of SIRT2 by cyclin-dependent kinase 1-dependent phosphorylation. J. Biol. Chem..

[15] Dong Z, Cui H (2016). Function of sirtuins in cancer stem cells. Int J. Stem Cell Res. Ther..

[16] Hiratsuka M (2003). Proteomics-based identification of differentially expressed genes in human gliomas: down-regulation of SIRT2 gene. Biochem. Biophys. Res. Commun..

[17] Kim HS (2011). SIRT2 maintains genome integrity and suppresses tumorigenesis through regulating APC/C activity. Cancer Cell.

[18] Park SH (2012). SIRT2 is a tumor suppressor that connects aging, acetylome, cell cycle signaling, and carcinogenesis. Transl. Cancer Res..

[19] Serrano L (2013). The tumor suppressor SirT2 regulates cell cycle progression and genome stability by modulating the mitotic deposition of H4K20 methylation. Genes Dev..

[20] Heltweg B (2006). Antitumor activity of a small-molecule inhibitor of human silent information regulator 2 enzymes. Cancer Res..

[21] Jin YH (2008). Sirt2 interacts with 14-3-3 beta/gamma and down-regulates the activity of p53. Biochem. Biophys. Res. Commun..

[22] Jing H (2016). A SIRT2-selective inhibitor promotes c-Myc oncoprotein degradation and exhibits broad anticancer activity. Cancer Cell.

[23] Zhang Y (2009). Identification of a small molecule SIRT2 inhibitor with selective tumor cytotoxicity. Biochem. Biophys. Res. Commun..

[24] Cha Y (2017). Metabolic control of primed human pluripotent stem cell fate and function by the miR-200c-SIRT2 axis. Nat. Cell Biol..

[25] Nichols J, Smith A (2009). Naive and primed pluripotent states. Cell Stem. Cell.

[26] Kurosawa H (2007). Methods for inducing embryoid body formation: in vitro differentiation system of embryonic stem cells. J. Biosci. Bioeng..

[27] Schorl C, Sedivy JM (2007). Analysis of cell cycle phases and progression in cultured mammalian cells. Methods.

[28] Li H (2009). The Ink4/Arf locus is a barrier for iPS cell reprogramming. Nature.

[29] Liao J (2008). Enhanced efficiency of generating induced pluripotent stem (iPS) cells from human somatic cells by a combination of six transcription factors. Cell Res..

[30] Tanimura N, Saito M, Ebisuya M, Nishida E, Ishikawa F (2013). Stemness-related factor Sall4 interacts with transcription factors Oct-3/4 and Sox2 and occupies Oct-Sox elements in mouse embryonic stem cells. J. Biol. Chem..

[31] Rais Y (2013). Deterministic direct reprogramming of somatic cells to pluripotency. Nature.

[32] Utikal J (2009). Immortalization eliminates a roadblock during cellular reprogramming into iPS cells. Nature.

[33] Unternaehrer JJ (2014). The epithelial-mesenchymal transition factor SNAIL paradoxically enhances reprogramming. Stem Cell Rep..

[34] Brambrink T (2008). Sequential expression of pluripotency markers during direct reprogramming of mouse somatic cells. Cell Stem. Cell.

[35] Si X (2013). Activation of GSK3beta by Sirt2 is required for early lineage commitment of mouse embryonic stem cell. PLoS ONE.

[36] Gomes P, Outeiro TF, Cavadas C (2015). Emerging role of sirtuin 2 in the regulation of mammalian metabolism. Trends Pharmacol. Sci..

[37] Maxwell MM (2011). The Sirtuin 2 microtubule deacetylase is an abundant neuronal protein that accumulates in the aging CNS. Hum. Mol. Genet..

[38] Nguyen P, Lee S, Lorang-Leins D, Trepel J, Smart DK (2014). SIRT2 interacts with beta-catenin to inhibit Wnt signaling output in response to radiation-induced stress. Mol. Cancer Res..

[39] Rittling SR, Denhardt DT (1992). p53 mutations in spontaneously immortalized 3T12 but not 3T3 mouse embryo cells. Oncogene.

[40] Rizzo M (2011). Immortalization of MEF is characterized by the deregulation of specific miRNAs with potential tumor suppressor activity. Aging.

[41] Gruber AJ (2014). Embryonic stem cell-specific microRNAs contribute to pluripotency by inhibiting regulators of multiple differentiation pathways. Nucleic Acids Res..

[42] Dryden SC, Nahhas FA, Nowak JE, Goustin AS, Tainsky MA (2003). Role for human SIRT2 NAD-dependent deacetylase activity in control of mitotic exit in the cell cycle. Mol. Cell Biol..

[43] Bae NS, Swanson MJ, Vassilev A, Howard BH (2004). Human histone deacetylase SIRT2 interacts with the homeobox transcription factor HOXA10. J. Biochem..

[44] Ivanchuk SM, Mondal S, Dirks PB, Rutka JT (2001). The INK4A/ARF locus: role in cell cycle control and apoptosis and implications for glioma growth. J. Neurooncol..

[45] Sherr CJ (2012). Ink4-Arf locus in cancer and aging. Wiley Interdiscip. Rev. Dev. Biol..

[46] Deng W (2015). MicroRNA replacing oncogenic Klf4 and c-Myc for generating iPS cells via cationized pleurotus eryngii polysaccharide-based nanotransfection. ACS Appl. Mater. Interfaces.

[47] Gonzalez S, Serrano M (2006). A new mechanism of inactivation of the INK4/ARF locus. Cell Cycle.

[48] Hong H (2009). Suppression of induced pluripotent stem cell generation by the p53-p21 pathway. Nature.

[49] Kawamura T (2009). Linking the p53 tumour suppressor pathway to somatic cell reprogramming. Nature.

[50] McCarthy AR (2013). Tenovin-D3, a novel small-molecule inhibitor of sirtuin SirT2, increasesp21 (CDKN1A) expression in a p53-independent manner. Mol. Cancer Ther..

[51] Kilic Eren M, Kilincli A, Eren O (2015). Resveratrol induced premature senescence is associated with DNA damage mediated SIRT1 and SIRT2 down-regulation. PLoS ONE.

[52] Yang H, Ye D, Guan KL, Xiong Y (2012). IDH1 and IDH2 mutations in tumorigenesis: mechanistic insights and clinical perspectives. Clin. Cancer Res..

[53] Fernandez-Arroyo S (2015). Activation of the methylation cycle in cells reprogrammed into a stem cell-like state. Oncoscience.

[54] Kwon OS, Han MJ, Cha HJ (2017). Suppression of SIRT2 and altered acetylation status of human pluripotent stem cells: possible link to metabolic switch during reprogramming. BMB Rep..

[55] Millson SH (2007). Expressed as the sole Hsp90 of yeast, the alpha and beta isoforms of human Hsp90 differ with regard to their capacities for activation of certain client proteins, whereas only Hsp90beta generates sensitivity to the Hsp90 inhibitor radicicol. FEBS J..

[56] Bradley E, Bieberich E, Mivechi NF, Tangpisuthipongsa D, Wang G (2012). Regulation of embryonic stem cell pluripotency by heat shock protein 90. Stem Cells.

[57] Ichimura T (2013). 14-3-3 proteins sequester a pool of soluble TRIM32 ubiquitin ligase to repress autoubiquitylation and cytoplasmic body formation. J. Cell Sci..

[58] Bahnassawy L (2015). TRIM32 modulates pluripotency entry and exit by directly regulating Oct4 stability. Sci. Rep..

[59] Hossini AM (2016). PI3K/AKT Signaling Pathway Is Essential for Survival of Induced Pluripotent Stem Cells. PLoS ONE.

[60] Qin H (2012). Transcriptional analysis of pluripotency reveals the Hippo pathway as a barrier to reprogramming. Hum. Mol. Genet..

