# Supplementary material for: SIRT2 is required for efficient reprogramming of mouse embryonic fibroblasts toward pluripotency
Source: Cell Death Dis. 2018 Aug 30;9(9):893. doi: 10.1038/s41419-018-0920-3 (PMC6117269; doi:10.1038/s41419-018-0920-3)
Supplement: Supplementary file 1 — Supplemental informations [file 41419_2018_920_MOESM1_ESM.doc]

**Title pages**

**Title:** SIRT2 is required for efficient reprogramming of mouse embryonic fibroblasts towards pluripotency

**Running title:** SIRT2 depletion hampers mouse iPSC generation

**Author names and affiliations:** Ah-Young Kim^1,2^, Eun-Mi Lee^1,2^, Eun-Joo Lee^1,2^, Jae-Hong Kim^3^, Kyoungho Suk^3^, Eunhye Lee^4^, Keun Hur^4^, Yean Ju Hong^5^, Jeong Tae Do^5^, SunYoung Park^1,2^ and Kyu-Shik Jeong^1,2,*^

^1^Department of Pathology, College of Veterinary Medicine, Kyungpook National University, Daegu 41566, Republic of Korea

^2^Stem Cell Therapeutic Research Institute, Kyungpook National University, Daegu 41566, Republic of Korea

^3^Department of Phamacology, Brain Science & Engineering Institute, BK21 Plus KNU Biomedical Convergence Program for Creative Talent, School of Medicine, Kyungpook National University, Daegu 41944, Republic of Korea

^4^Department of Biochemistry and Cell Biology; and BK21 Plus KNU Biomedical Convergence Program for Creative Talent, Department of Biomedical Science, School of Medicine, Kyungpook National University, Daegu 41944, Republic of Korea

^5^Department of Stem Cells and Regenerative Biology, College of Animal Bioscience and Technology, Konkuk University, Seoul 05029, Republic of Korea

***Corresponding author:** Kyu-Shik Jeong, D.V.M., Ph.D.

Department of Pathology, College of Veterinary Medicine, Kyungpook National University, Daegu 702-701, Republic of Korea

Stem Cell Therapeutic Research Institute, Kyungpook National University, Daegu 702-701, Republic of Korea

Tel: 82-53-950-5975

Fax: 82-52-950-5955

E-mail: jeongks@knu.ac.k

**Supplemental Information**

**Inventory of Supplemental Information**

**Supplemental Figures and Legends:**

- **Figure S1 (refers to Figure 2)**
- **Figure S2 (refers to Figure 4)**
- **Figure S3 (refers to Figure 3 and Figure 4)**
- **Figure S4 (refers to Figure 5 and sFigure 5)**
- **Figure S5 (refers to Table 1)**

**Supplemental Table 1 (refers to experimental procedures)**

**Supplemental Table 2 (refers to experimental procedures)**

**Supplemental Table 3 (refers to supplemental experimental procedures)**

**Supplemental Experimental Procedures**

**Supplemental Reference**

**Figure S1. Identification of genotype and phenotype of SIRT2-WT/HT/KO MEFs.**


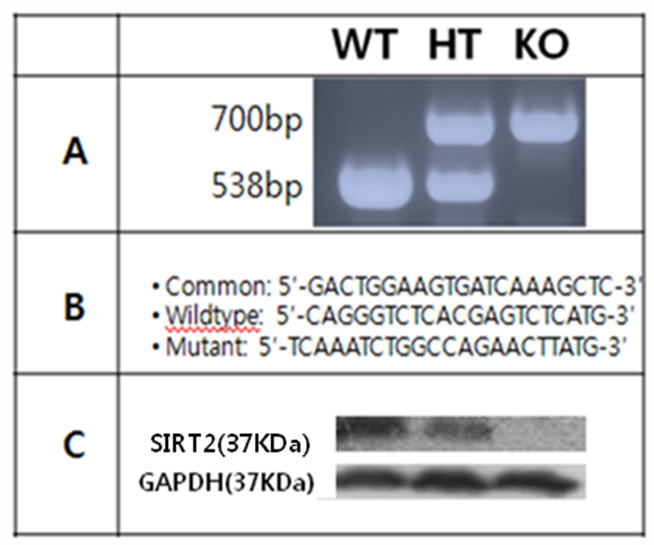


**Figure S1. Identification of genotype and phenotype of SIRT2-WT/HT/KO MEFs (related to Figure 2).**

(A) Genotypes were identified by PCR with genomic DNA. (B) Primers used in genotyping. (C) Phenotypes were confirmed by western blotting using anti-SIRT2 antibody (Sigma-Aldrich, St. Louis, MO, USA). GAHDP (Cell Signaling Technologies, Danvers, MA, USA) was used as a loading control.

**Figure S2. Frequent lethality during embryonic development of SIRT2-KO-iPSC chimeras**

**
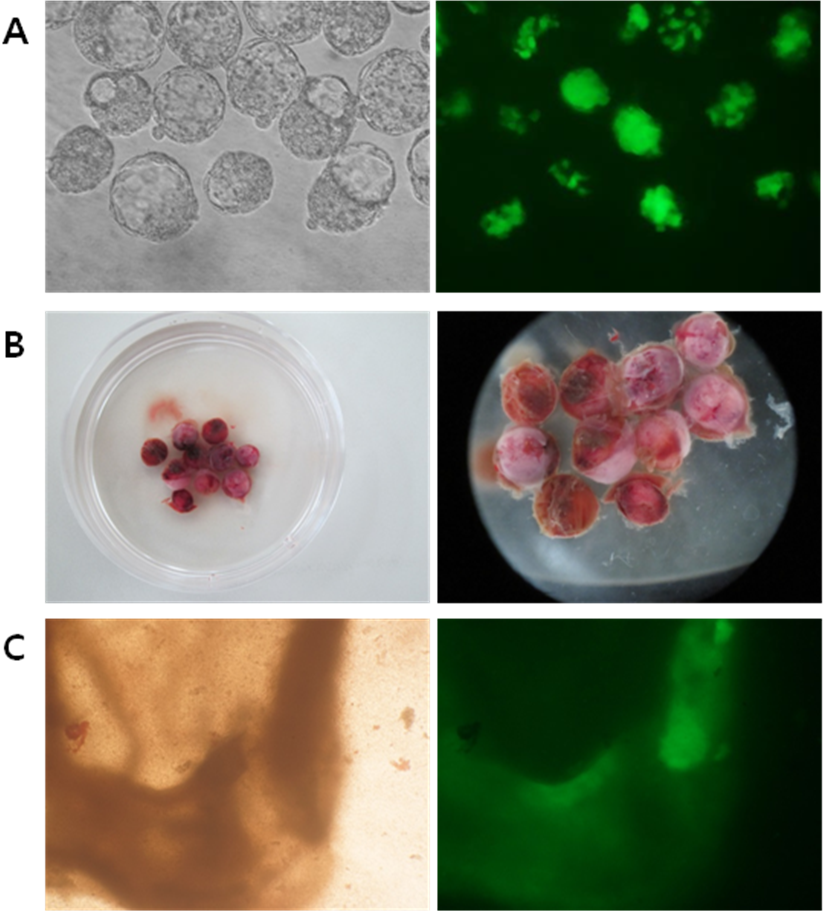
**

**Figure S2. Frequent lethality during embryonic development of SIRT2-KO-iPSC chimeras (related to Figure 4).**

(A) B6D2F1 blastocysts aggregated with EGFP-electroporated SIRT2-KO-iPSCs. (B) Frequent embryonic lethality during 10.5–11.5 dpc. (C) GFP-positive tissues were observed in the incompletely developed embryo.

**Figure S3. Methylation status of Oct4 and Nanog gene promoters**

**
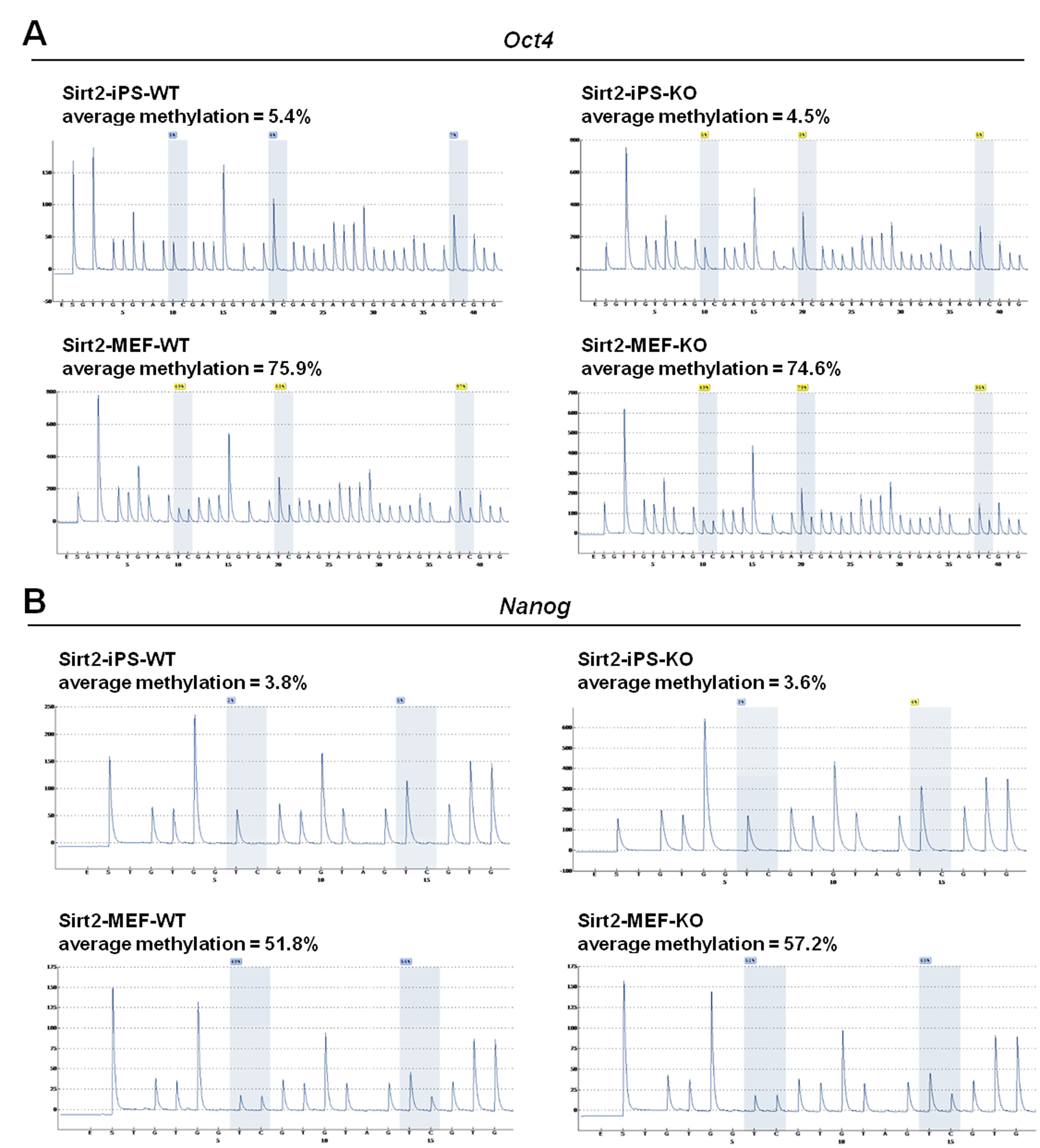
**

**Figure S3. Methylation status of Oct4 and Nanog gene promoters (related to Figure 3 and Figure 4).**

Methylation status of (A) Oct4 and (B) Nanog. Gray vertical boxes in the pyrograms illustrate individual CpG sites analyzed.

**Figure S4. Full-length images of western blot data and additional qRT-PCR data (Related to Figure 5 and Figure S1)**

1. **
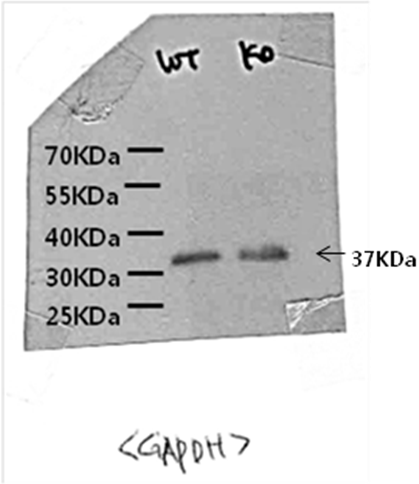
 (B)
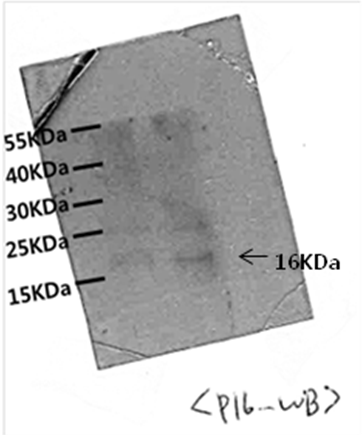
**

**(C)**
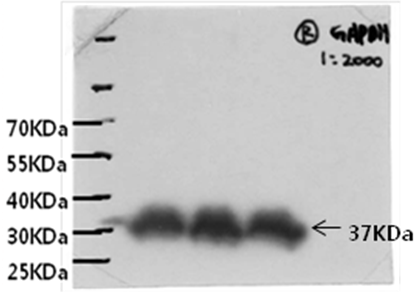
**(D)
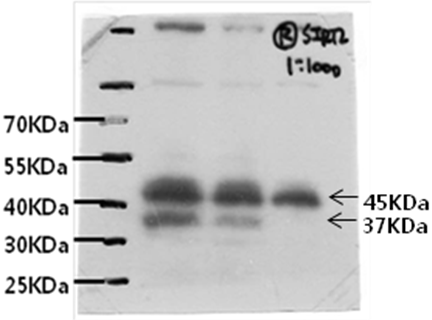
**

**
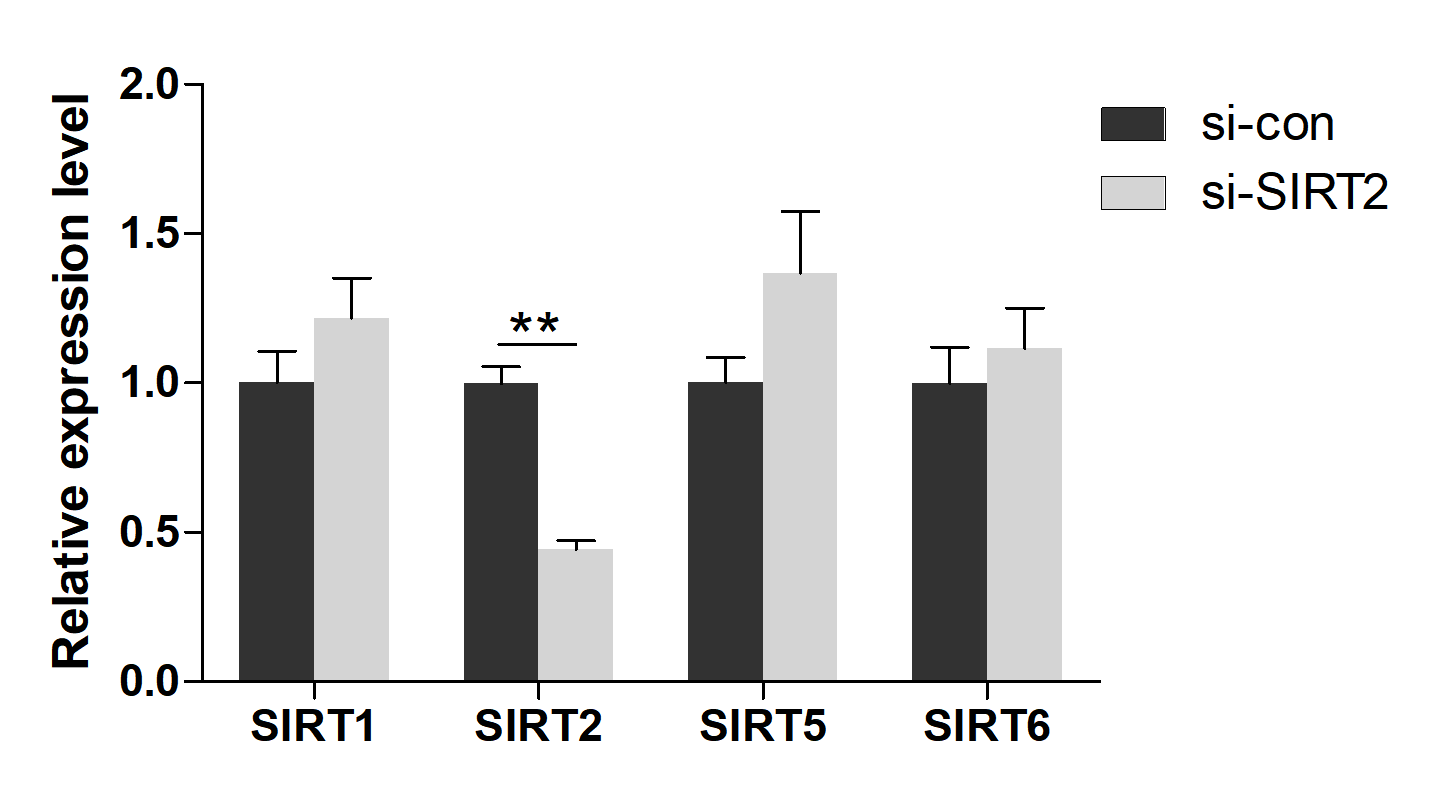

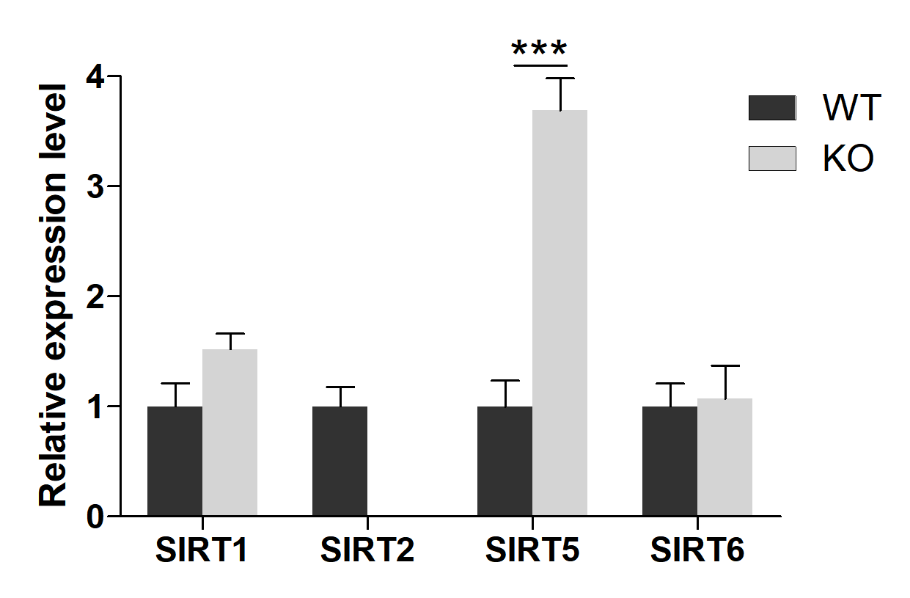
**

**(E) (F)**

**Figure S4. Full-length images of western blot data and additional qRT-PCR data (Related to Figure 5 and Figure S1)**

Respetive full-length image of Figure 5E, GAPDH (A), Figure 5E, P16^Ink4a^ (B), Figure S1, GAPDH (C), Figure S1, SIRT2 (D), and the RT-PCR data to show the impact of SIRT2-deficiency on the expression levels of SIRT1, SIRT2, SIRT5 and SIRT6 between si-control and si-SIRT2-transfected MEF (E), (n=3), and between WT and SIRT2 KO iPSCs (F), (n=4).

**Figure S5. Gene ontology and KEGG pathway analysis of differentially expressed proteins between wild-type and SIRT2-knockout MEFs (related to Table 1).**

**
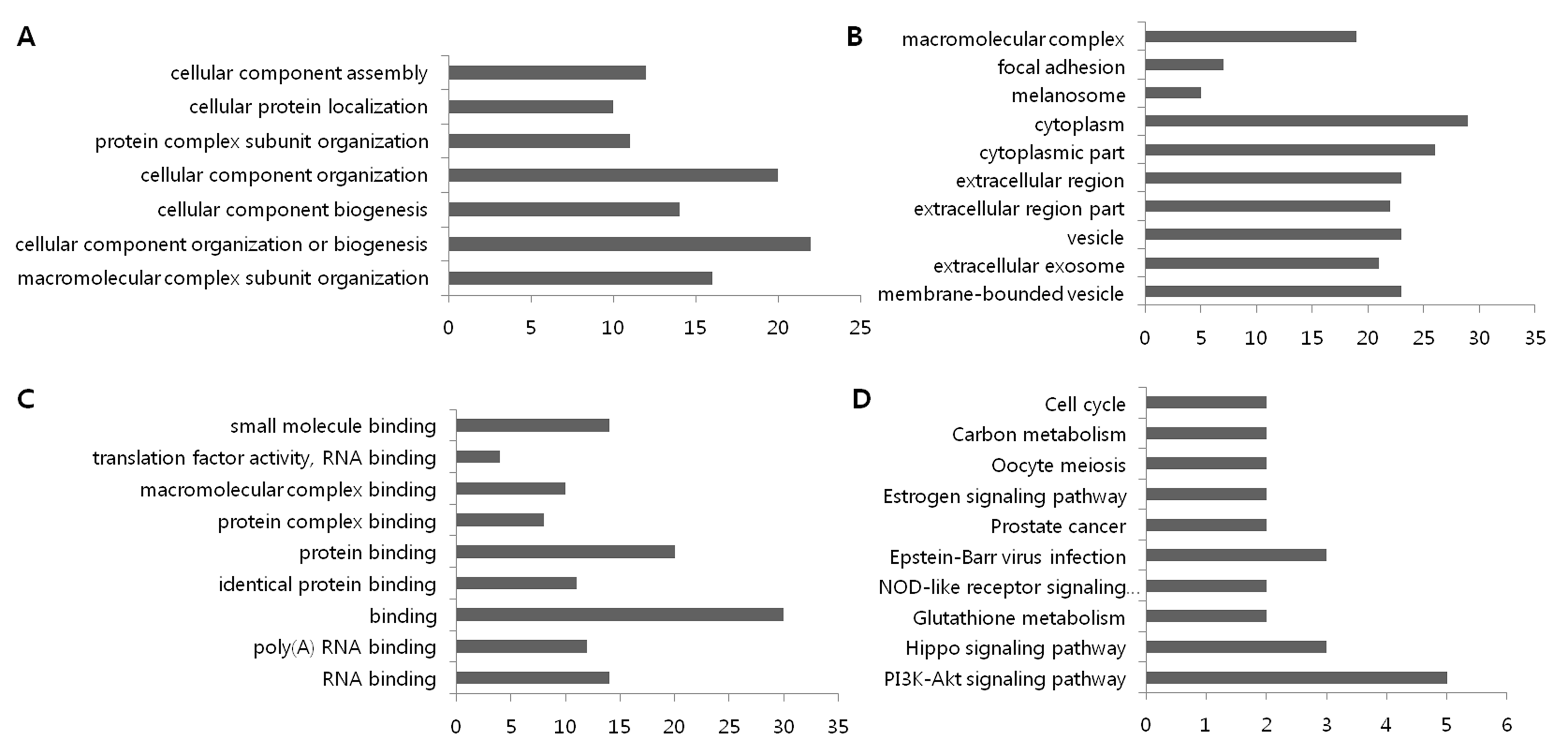
**

**Figure S5. Gene ontology and KEGG pathway analysis of differentially expressed proteins between wild-type and SIRT2 knockout MEFs (related to Table 1).**

(A) Biological process, (B) cellular component, (C) molecular function, and (D) pathway analysis are shown. Statistically significant data (P < 0.05) are shown.

Table S1. Semi-quantitative RT-PCR primers

| **Gene** | **Primer sequences (5ʹ→3ʹ)** |
| --- | --- |
| **Total Sirt2** | **F: TGGCCTGGCTGGGTGACTGT**  **R: GGCAGGCGGTGGGGACTTTC** |
| **Sirt2.1** | **F: AGGCTCAGGATTCAGACTCG**  **R: TGTAGCGTGTCACTCCTTCG** |
| **Sirt2.2** | **F: GAGCCGGACCGATTCAGAC**  **R: TTCGAGGGTCAGCTCGTCTA** |
| **Oct4** | **F: CTGAGGGCCAGGCAGGAGCACGAG**  **R: CTGTAGGGAGGGCTTCGGGCACTT** |
| **Rex1** | **F: CACCATCCGGGATGAAAGTGAGAT**  **R: ACGAGAAAATGTCGCTTTAGTTTC** |
| **Nestin** | **F: CCCTGAAGTCGAGGAGCTG**  **R: CTGCTGCACCTCTAAGCGA** |
| **Brachyury** | **F: GCTGGATTACATGGTCCCAAG**  **R: GGCACTTCAGAAATCGGAGGG** |
| **Sox17** | **F: GATGCGGGATACGCCAGTG**  **R: CCACCTCGCCTTTCACCTTTA** |
| **Gapdh** | **F: GGTCGGTGTGAACGGATTTG**  **R: TCGTTGATGGCAACAATCTCCACT** |

Table S2. Quantitative RT-PCR primers

| **Gene** | **Primer sequences (5ʹ→3ʹ)** |
| --- | --- |
| **Total Sirt2** | **F: ACTGGCCTCTATGCAAACCT**  **R: AGGGCAAAGAAGGGTTCCG** |
| **ALP** | **F: ACAACCTGACTGACCCTTCG**  **R: GGTCAATCCTGCCTCCTTCC** |
| **P15^ink4b^** | **F: TCAGAGACCAGGCTGTAGCAATC**  **R: CCCCGGTCTGTGGCAGAA** |
| **P16^ink4a^** | **F: CCCAACGCCCCGAACT**  **R: GTGAACGTTGCCCATCATCA** |
| **P19^arf^** | **F: GCCGCACCGGAATCCT**  **R: TTGAGCAGAAGAGCTGCTACGT** |
| **P21** | **F: TTCCGCACAGGAGCAAAGT**  **R: CGGCGCAACTGCTCACT** |
| **P53** | **F: AAGATCCGCGGGCGTAA**  **R: CATCCTTTAACTCTAAGGCCTCATTC** |
| **Rb** | **F: TCTACCTCCCTTGCCCTGTTT**  **R: CAGAAGGCGTGCACAGAGTGT** |
| **Sirt1** | **F: TCGTGGAGACATTTTTAATCAGG**  **R: GCTTCATGATGGCAAGTGG** |
| **Sirt2** | **F: AGGCTCAGGATTCAGACTCG**  **R: TGTAGCGTGTCACTCCTTCG** |
| **Sirt3** | **F: TCCTCTGAAACCGGATGG**  **R: TCCCACACAGAGGGATATGG** |
| **Sirt4** | **F: CCAAAGCAGGGAGTCAGC**  **R: GCAGTCTGCTCCCCACAG** |
| **Sirt5** | **F: CCAGCTTTAGCAGGAAAAGG**  **R: GACTGGGATTCTGGCGTCT** |
| **Sirt6** | **F: ACGCGGATAAGGGCAAGT**  **R: CTCCCACACCTTGCGTTC** |
| **Sirt7** | **F: TGCAACTCCTCATGAATGAACT**  **R: CGCCAAGGAGAAGATTGG** |
| **β-actin** | **F: GACGGCCAGGTCATCACTATTG**  **R: AGGAAGGCTGGAAAAGAGCC** |

Table S3. Primers for pyrosequencing analysis

| Gene | Primer sequence (5′–3′) | Product size (bp) |
| --- | --- | --- |
| *Oct4* | Forward: TGGTTGAGTGGGTTGTAAGGA | 163 |
|  | Reverse: biotin-AAAACCAAACCCCAAAAAACCTTCATT |  |
|  | Sequencing: GGGTGTAGTGTTAATAGG |  |
| *Nanog* | Forward: TGGTGGATTTTGTAGGTGGGATTAATTG | 97 |
|  | Reverse: biotin-TACCCTACCCACCCCCTATT |  |
|  | Sequencing: ATTGTGAATTTATAGGGTTG |  |

**Supplemental Experimental Procedures**

Animals

Wild-type (WT) C57BL/6 mice (JAX stock #000664) and SIRT2 knockout (KO) mice (JAX stock #012772) were purchased from the Jackson Laboratory (Bar Harbor, ME, USA). They were housed and bred in a room at 22 ± 3°C with a relative humidity of 50 ± 10%, 12-h light-dark cycle, and *ad libitum* access to food and water. SIRT2 hetero (HT) mice were produced by crossing the WT C57BL/6 mice and SIRT2 KO mice, and were also housed and bred as described above. Genomic DNA was purified from mouse-tail tissue and then genotyping of each type of mouse was performed by PCR using a forward primer (5′-GACTGGAAGTGATCAAAGCTC-3′), WT-specific reverse primer (5′-CAGGGTCTCACGAGTCTCATG-3′), and KO-specific reverse primer (5′-TCAAATCTGGCCAGAACTTATG-3′). Animal experiments were performed in accordance with the NIH Guide for the Care and Use of Laboratory Animals and approved by the Institutional Animal Care and Use Committee of Kyungpook National University (KNU 2011-0019 and KNU 2014-0167).

DNA methylation analysis

Genomic DNA was extracted from four cell lines (Sirt2-iPSC-WT, Sirt2-iPSC-KO, Sirt2-MEF-WT, and Sirt2-MEF-KO) using the QIAmp DNA Mini Kit (Qiagen, Hilden, Germany). Bisulfite modification was performed with 1 μg of extracted genomic DNA using the EZ DNA Methylation™-GOLD Kit (Zymo Research, Irvine, CA, USA) following the manufacturer’s instructions. Bisulfite PCR and pyrosequencing primers were designed to amplify target regions of the Oct4 and Nanog genes using PSQ Assay Design software (Qiagen). Sequences of the primers used are shown in Table S3. Methylation levels of Oct4 and Nanog genes were analyzed by quantitative bisulfite pyrosequencing using the PyroMark Q24 system (Qiagen) following the manufacturer’s instructions.

2-DE

2-DE was carried out essentially as described previously (Park et al., 2002). Aliquots in sample buffer (7 M urea, 2 M thiourea, 4.5% CHAPS, 100 mM DTE, and 40 mM Tris, pH 8.8) were applied to immobilized pH 3–10 nonlinear gradient strips (Amersham Biosciences, Amersham, UK). IEF was performed at 80,000 Vh. The second dimension was analyzed on 9–16% linear gradient polyacrylamide gels (18 cm × 20 cm × 1.5 mm) at constant 40 mA per gel for approximately 5 h. After protein fixation in 40% methanol and 5% phosphoric acid for 1 h, the gels were stained with CBB G-250 for 12 h. The gels were destained with H_2_O, scanned in a Bio-Rad GS710 densitometer (Richmond, CA), and then converted into electronic files, which were then analyzed using the Image Master Platinum 5.0 image analysis program (Amersham Biosciences).

In-gel tryptic digestion

The spots of interest for analysis were excised from the preparative gel and then individually transferred into 1.5-mL tubes. The spots were washed with 100 μL distilled water, followed by addition of 100 μL 50 mM NH_4_HCO_3_ (pH 7.8) and acetonitrile (6:4) and shaking for 10 min. This process was repeated at least three times until the Coomassie brilliant blue G250 dye disappeared. The supernatant was decanted, the band was dried in a speed vacuum concentrator (LaBoGeneAps, Lynge, Denmark) for 10 min, and then digested with sequence-grade modified trypsin (enzyme to substrate ratio, 1:30; Promega, Madison, WI, USA) at 37°C with shaking for 16 h.

LC-MS/MS for peptides analysis

Nano LC-MS/MS analysis was performed with an Easy n-LC (Thermo Fisher Scientific, Waltham, MA, USA) and LTQ Orbitrap XL mass spectrometer (Thermo Fisher Scientific) equipped with a nano-electrospray source. Samples were separated on A C18 nanobore columns (150 mm × 0.1 mm and 3 μm pore size; Agilent Technologies, Santa Clara, CA, USA). Mobile phase A for LC separation was 0.1% formic acid and 3% acetonitrile in deionized water, while mobile phase B was 0.1% formic acid in acetonitrile. The chromatography gradient was designed for a linear increase from 5% B to 30% B in 23 min, 30% B to 60% B in 3 min, 95% B in 3 min, and 3% B in 6 min. The flow rate was maintained at 1500 nL/min. Mass spectra were acquired using data-dependent acquisition with a full mass scan (350–1200 m/z) followed by 10 MS/MS scans. For MS1 full scans, the orbitrap resolution was 15,000 and the AGC was 2 × 10^5^. For MS/MS using the LTQ, the AGC was 1 × 10^4^.

Database searching

The mascot algorithm (Matrix Science, Boston, MA, USA) was used to identify peptide sequences present in a protein sequence database. Database search criteria were as follows, taxonomy, *Mus musculus* (downloaded May 22 2013, 25805290 sequences); fixed modification, carbamidomethylated at cysteine residues; variable modification, oxidized at methionine residues, acetylated at N-terminal and lysine residues, or deamidated at asparagine and glutamine residues; maximum allowed missed cleavage, 2; MS tolerance, 10 ppm; MS/MS tolerance, 0.8 Da. Peptides were filtered with a significance threshold of P < 0.05.

Gene silencing using siRNA

For SIRT2 gene silencing, 60% confluent MEF cells were pre-incubated with High-glucose DMEM (Thermo Fisher Scientific, MA, USA) containing 10% fetal bovine serum (Thermo Fisher Scientific), 1% MEM-NEAA (Thermo Scientific), 1% GlutaMAX (Thermo Fisher Scientific) and 10 mM β-mercaptoethanol (Amnesco, OH, USA) without penicillin/streptomycin (Thermo Fisher Scientific) for 1 h and transfected with 100 nM of SIRT2 small interfering RNA (siRNA) (5’-ACCUGGAGAAGUACCACCUUCCUUA-3′, Bioneer, Daegeon, Korea) or scrambled siRNA (SN-1002, Bioneer, Daejeon, Korea) using Lipofectamine 2000 (Life Technologies, Carlsbad, CA, USA) for 48 h.

**Supplemental Reference**

Park KS, Kim H, Kim NG, Cho SY, Choi KH, Seong JK, *et al*. Proteomic alterations of the variants of human aldehyde dehydrogenase isozymes correlate with hepatocellular carcinoma. *Hepatology* 2002; **35**: 1459-1466.
